# Silage-Based Forages: From Fermentation Quality to Ruminant Health and Sustainable Production

**DOI:** 10.3390/ani16081214

**Published:** 2026-04-16

**Authors:** Fulin Yang

**Affiliations:** College of Animal Sciences, Fujian Agriculture and Forestry University, Fuzhou 350002, China; fulin.yang@fafu.edu.cn

## 1. Introduction

Efficient utilization of roughage is central to ruminant nutrition, animal health, and the environmental footprint of livestock systems. Silage, as a key technology for forage preservation, directly influences rumen function, animal performance, and production sustainability. This Special Issue, Impacts of silage-based forages on ruminant health and welfare, presents five studies that collectively advance our understanding of silage-based feeding strategies, spanning fermentation optimization, novel forage resources, and their impacts on animal productivity and methane mitigation.

## 2. The Structure of This Special Issue

### 2.1. Optimizing Silage Quality: Additives and Forage Characteristics

Three studies in this issue explore how inoculants and agronomic practices shape silage fermentation and nutritional value.

Chagas et al. [Contribution 1] evaluated *Azospirillum brasilense* inoculation combined with nitrogen fertilization in giant sorghum silage under Brazilian Amazon conditions. Inoculation did not improve agronomic traits and reduced first-cut yield at 200 and 400 kg N/ha, with total yield 13.0% lower than non-inoculated controls (19.24 vs. 22.12 t/ha). Although inoculation slightly increased crude protein (7.67% vs. 7.01%), the authors concluded that, under these edaphoclimatic conditions, 200 kg N/ha without inoculation was the most effective strategy.

Jin et al. [Contribution 2] compared *Lactiplantibacillus plantarum* and *Lentilactobacillus buchneri* in Silphium perfoliatum silage. After 60 days, both inoculants increased crude protein (19.29 vs. 15.53–15.87 g/kg DM in controls). *L. buchneri* resulted in higher acetic acid, lower ammonia nitrogen, and superior aerobic stability, while *L. plantarum* produced more lactic acid. *L. buchneri* also improved in vitro crude protein digestibility (57.80% vs. 54.77–55.77%). The study recommends *L. buchneri* as the preferred additive for this forage.

Lelis et al. [Contribution 3] assessed commercial inoculants and fermented milk (Yakult^®^) in BRS Capiaçu elephant grass silage harvested at 90 or 105 days of regrowth. Kera-Sil produced the lowest pH (3.70) and lowest ammonia nitrogen (5.77% of total nitrogen), while Yakult^®^ resulted in the highest ammonia nitrogen (11.18%). At 90 days of regrowth, Kera-Sil and Silo-Max increased dry matter content and in vitro dry matter digestibility. The authors do not recommend Yakult^®^ for elephant grass silage.

### 2.2. From Silage to Rumen: Impacts on Animal Performance

Two studies in this issue provide integrated evidence linking silage quality to rumen function and production outcomes.

Olorunlowu et al. [Contribution 4] evaluated a multi-strain inoculant (*L. plantarum*, *L. buchneri*, *Propionibacterium acidipropionici*, *P. thoeni*) in dairy cows. Inoculated silage had lower pH (4.56 vs. 5.06) and higher crude protein (129 vs. 111 g/kg DM). Cows fed inoculated silage showed increased ruminal propionate (28.3 vs. 26.3 mM), reduced ammonia (7.61 vs. 8.67 mM), and lower protozoa counts (1.21 vs. 1.66 × 10^5^/mL). Methane emissions decreased both per cow (368 vs. 397 g/d) and per kg dry matter intake (15.1 vs. 16.5 g/kg DMI). Milk yield also increased, demonstrating that improved silage quality can simultaneously enhance productivity and reduce environmental impact.

Wang et al. [Contribution 5] examined replacing corn silage with sweet sorghum silage (forage or sugar type) in Boer goats. The 50% substitution level was most effective: average daily gain increased by 27.6% (forage sorghum) and slaughter rate reached 47.3% (sugar sorghum), a 12.4% improvement over controls. Meat from the 50% sugar sorghum group showed 24.7% higher crude protein and 5.0% higher crude fat. Unsaturated fatty acids increased by 11.9–15.6% across treatment groups. Rumen microbial analysis revealed that the relative abundance of Ruminococcus—a key fiber-degrading genus—rose from 15.63% in controls to 32.12–35.26% in sorghum silage groups, while Prevotella decreased from 28.56% to 23.21–25.63%.

### 2.3. Synthesis

Collectively, these five studies underscore that silage quality is not an endpoint but a critical determinant of rumen function, animal production efficiency, and environmental sustainability. Olorunlowu et al. [Contribution 4] provide direct evidence that silage inoculants can reduce methane emissions by 29 g/d per cow. Wang et al. [Contribution 5] demonstrate that drought-tolerant sweet sorghum can effectively substitute corn silage while improving growth performance and meat quality. Lelis et al. [Contribution 3] and Jin et al. [Contribution 2] offer region-specific recommendations for tropical and unconventional forages, emphasizing that inoculant efficacy depends on both crop species and microbial strain. Chagas et al. [Contribution 1], in turn, caution that microbial inoculation must be validated under local conditions, as benefits observed elsewhere may not translate universally.

## 3. Conclusions and Future Directions

Together, these studies reaffirm that strategic management of silage-based forages is a powerful lever for improving ruminant productivity and sustainability. Looking forward, deeper integration of microbiome analyses with animal performance metrics will help elucidate the mechanistic links between silage fermentation, rumen ecology, and host physiology. Expanding the evaluation of alternative forages across diverse agroecological zones will further support sustainable intensification. Finally, incorporating environmental indicators (e.g., methane emissions, nitrogen efficiency) into silage evaluation frameworks will align livestock production with global climate goals.

